# Supplementary material for: Climate Overrides the Influence of Microsite Conditions on Radial Growth of the Tall Multi-Stemmed Shrub Alnus alnobetula at Treeline
Source: Plants (Basel). 2023 Apr 20;12(8):1708. doi: 10.3390/plants12081708 (PMC10143859; doi:10.3390/plants12081708)
Supplement: Supplementary file 1 [file plants-12-01708-s001.zip › plants-2313117-supplementary.pdf]

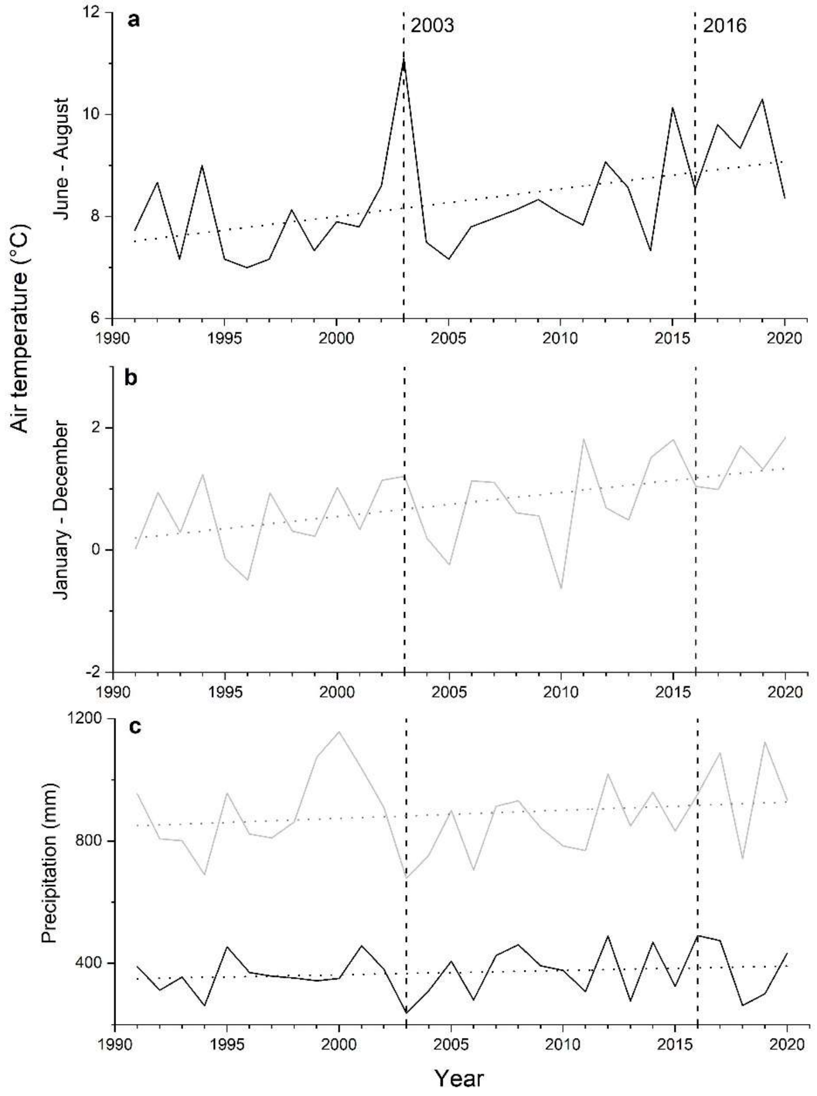

Figure S1. Mean air temperature (**a**, **b**) and precipitation (**c**) during June–August (black lines) and January–December (grey lines) recorded during 1991–2020 at Mt. Patscherkofel (2246 m asl). Linear air temperature and precipitation trends are shown in dotted lines. Extreme growth years (2003 and 2016) are indicated by vertical dashed lines.

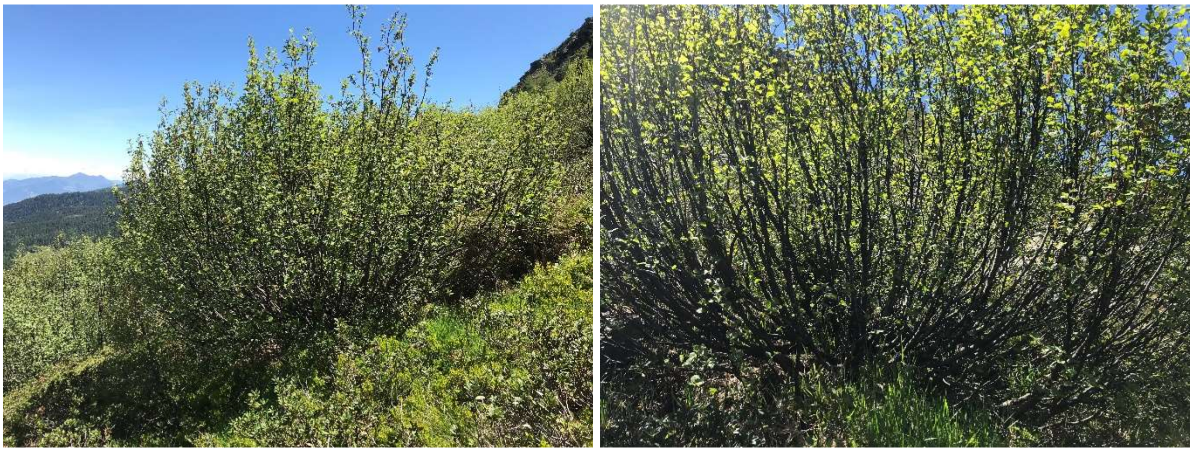

Figure S2. Green alder (*Alnus alnobetula*) individual located within an avalanche gully (site AG). Canopy height and stock diameter amount to c. 2.5 and 3 m, respectively.
